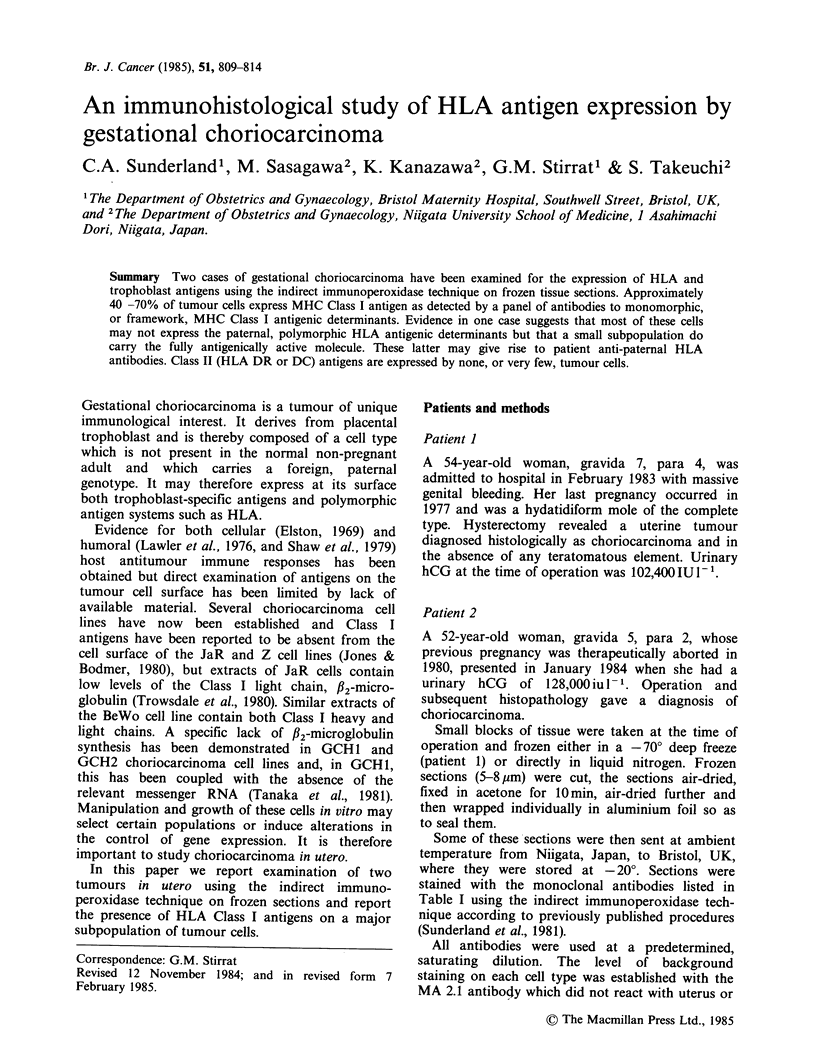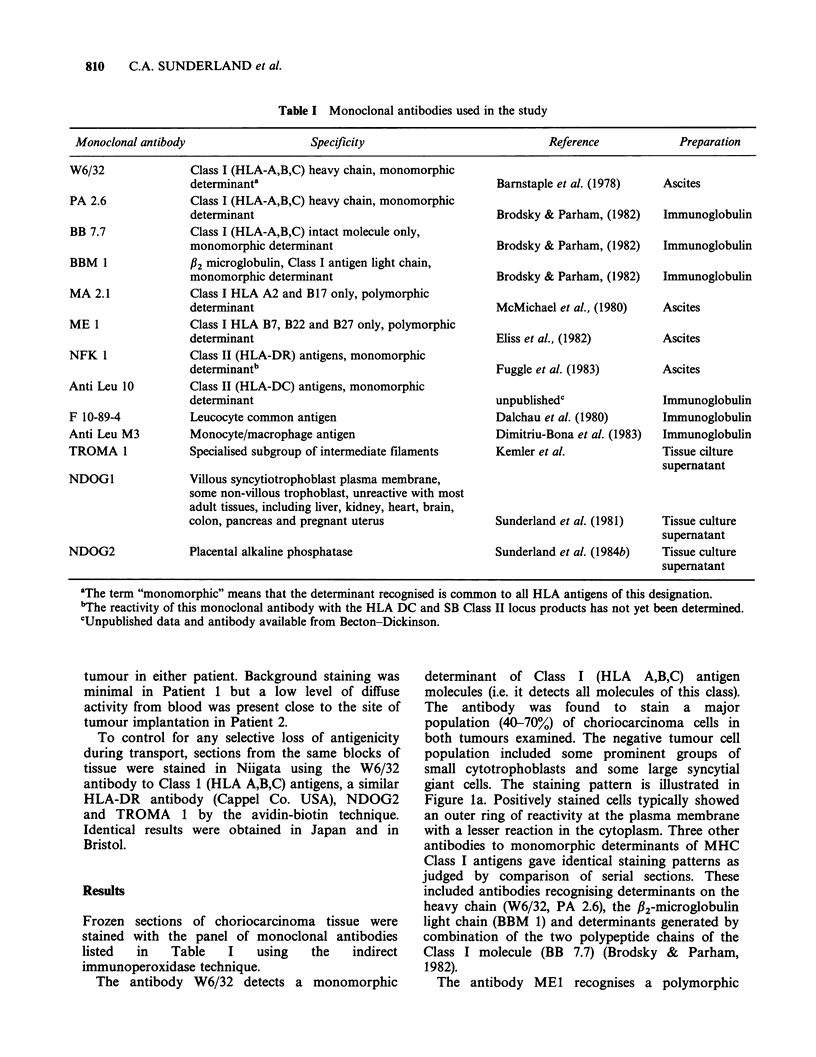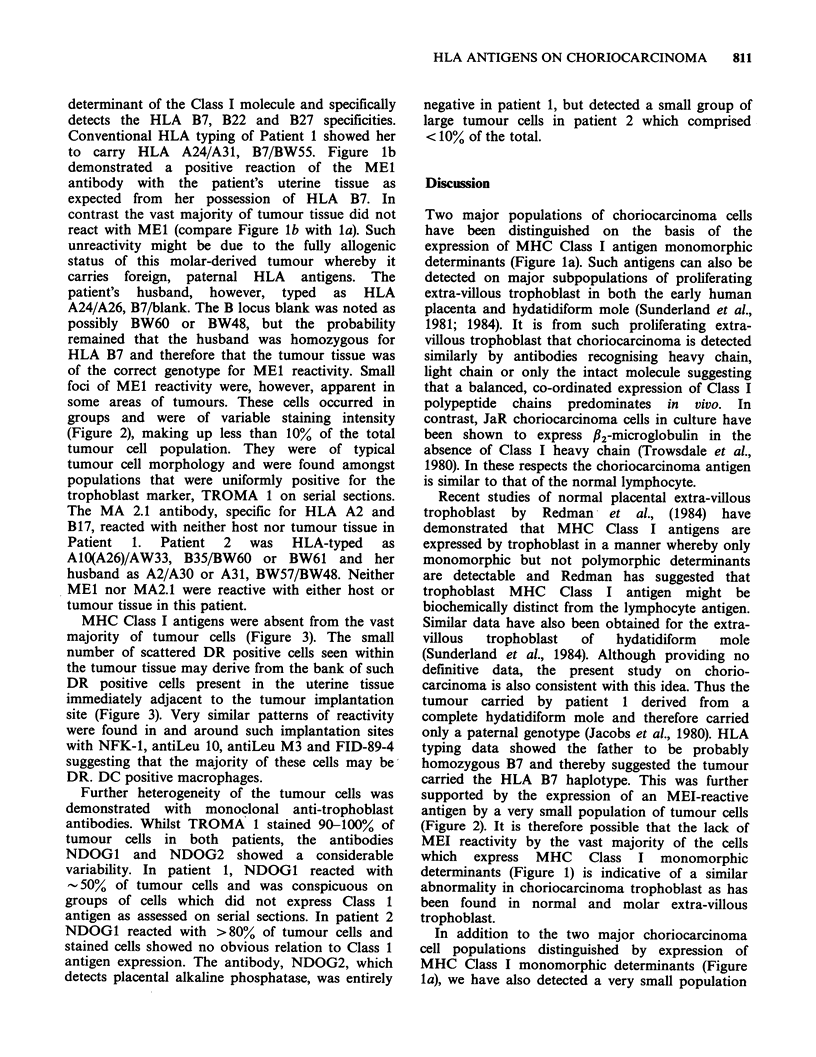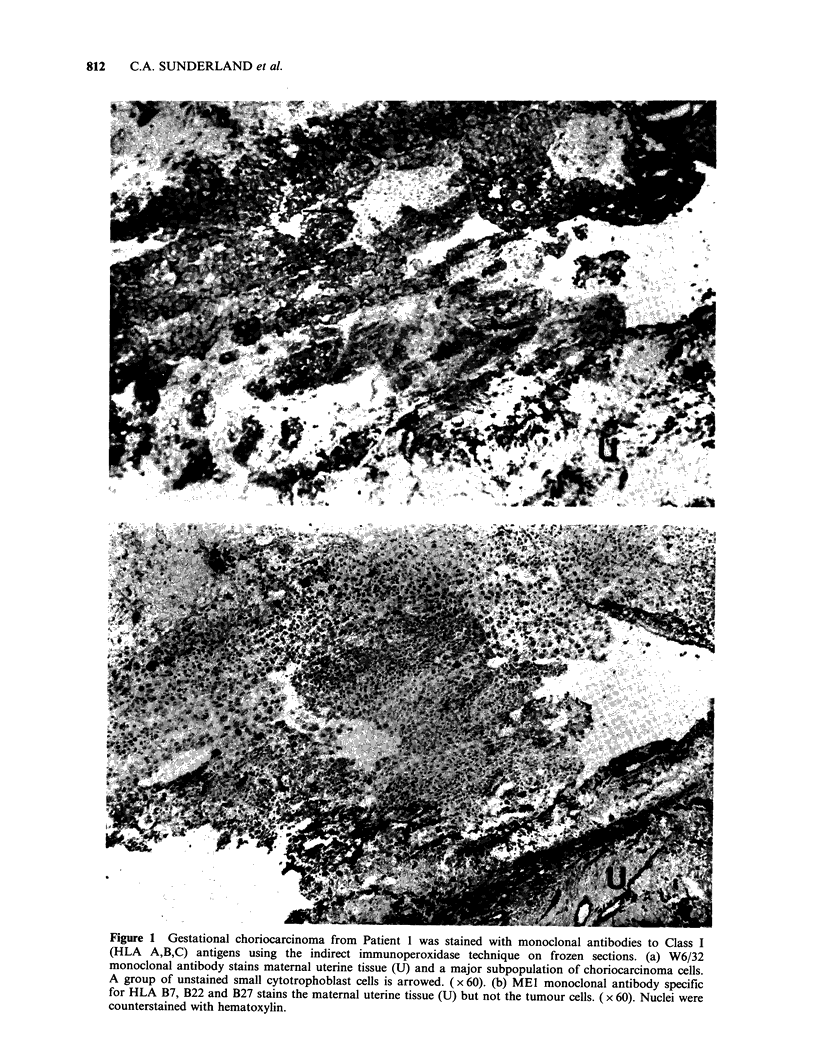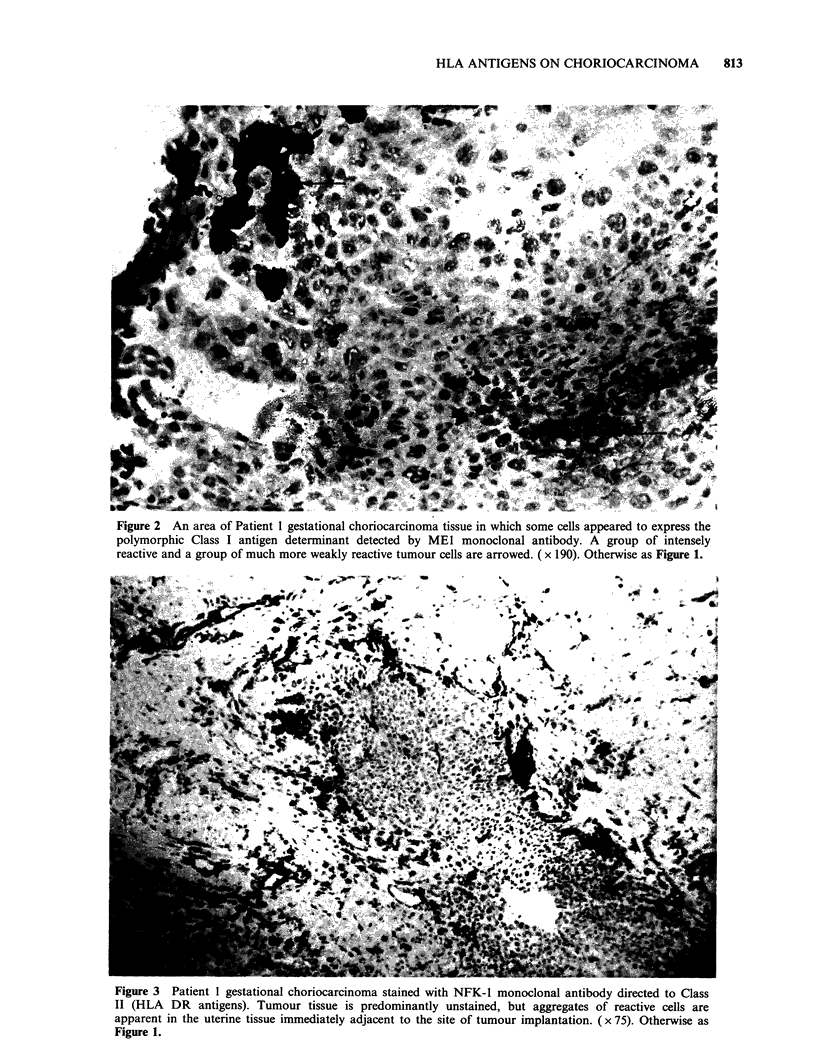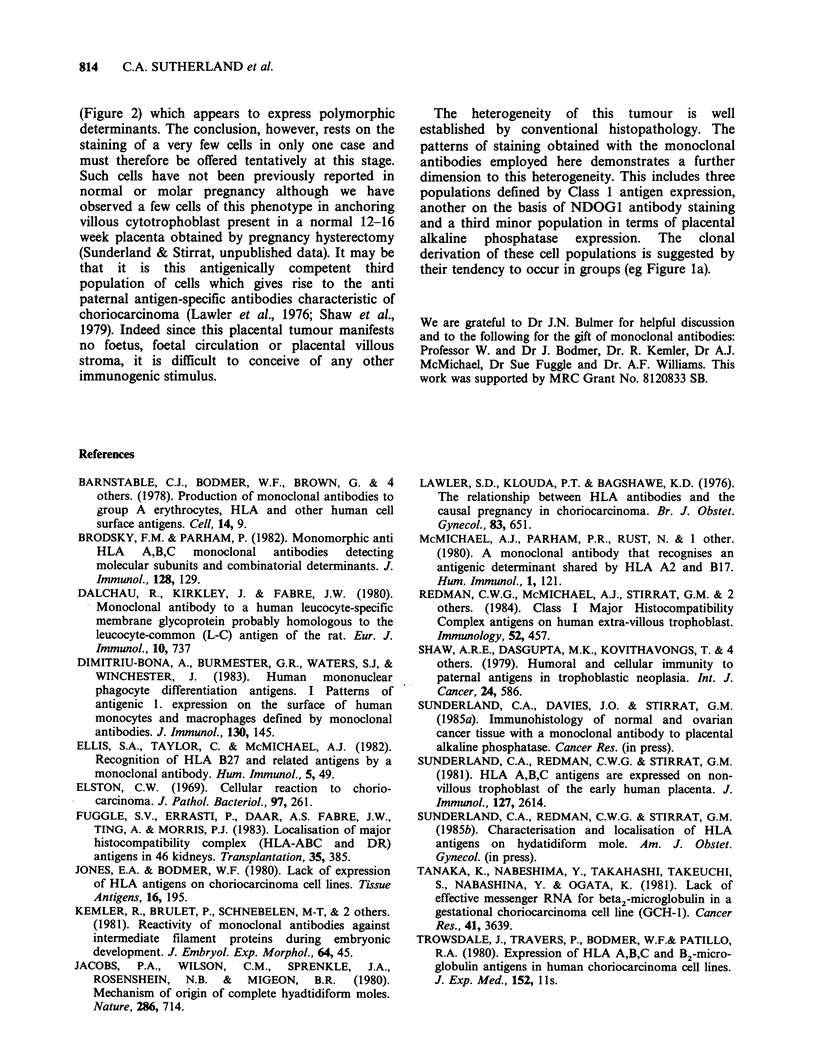# An immunohistological study of HLA antigen expression by gestational choriocarcinoma.

**DOI:** 10.1038/bjc.1985.126

**Published:** 1985-06

**Authors:** C. A. Sunderland, M. Sasagawa, K. Kanazawa, G. M. Stirrat, S. Takeuchi

## Abstract

**Images:**


*Br. J. Cancer* (1985), **51**, 809–814

### An immunohistological study of HLA antigen expression by gestational choriocarcinoma

C.A. Sunderland[1], M. Sasagawa[2], K. Kanazawa[2], G.M. Stirrat[1] & S. Takeuchi[2]

[1]*The Department of Obstetrics and Gynaecology, Bristol Maternity Hospital, Southwell Street, Bristol, UK, and* [2]*The Department of Obstetrics and Gynaecology, Niigata University School of Medicine, 1 Asahimachi Dori, Niigata, Japan.*

**Summary** Two cases of gestational choriocarcinoma have been examined for the expression of HLA and trophoblast antigens using the indirect immunoperoxidase technique on frozen tissue sections. Approximately 40 –70% of tumour cells express MHC Class I antigen as detected by a panel of antibodies to monomorphic, or framework, MHC Class I antigenic determinants. Evidence in one case suggests that most of these cells may not express the paternal, polymorphic HLA antigenic determinants but that a small subpopulation do carry the fully antigenically active molecule. These latter may give rise to patient anti-paternal HLA antibodies. Class II (HLA DR or DC) antigens are expressed by none, or very few, tumour cells.

Gestational choriocarcinoma is a tumour of unique immunological interest. It derives from placental trophoblast and is thereby composed of a cell type which is not present in the normal non-pregnant adult and which carries a foreign, paternal genotype. It may therefore express at its surface both trophoblast-specific antigens and polymorphic antigen systems such as HLA.

Evidence for both cellular (Elston, 1969) and humoral (Lawler *et al.*, 1976, and Shaw *et al.*, 1979) host antitumour immune responses has been obtained but direct examination of antigens on the tumour cell surface has been limited by lack of available material. Several choriocarcinoma cell lines have now been established and Class I antigens have been reported to be absent from the cell surface of the JaR and Z cell lines (Jones & Bodmer, 1980), but extracts of JaR cells contain low levels of the Class I light chain, $\beta_2$-microglobulin (Trowsdale *et al.*, 1980). Similar extracts of the BeWo cell line contain both Class I heavy and light chains. A specific lack of $\beta_2$-microglobulin synthesis has been demonstrated in GCH1 and GCH2 choriocarcinoma cell lines and, in GCH1, this has been coupled with the absence of the relevant messenger RNA (Tanaka *et al.*, 1981). Manipulation and growth of these cells *in vitro* may select certain populations or induce alterations in the control of gene expression. It is therefore important to study choriocarcinoma *in utero*.

In this paper we report examination of two tumours *in utero* using the indirect immunoperoxidase technique on frozen sections and report the presence of HLA Class I antigens on a major subpopulation of tumour cells.

Correspondence: G.M. Stirrat
Revised 12 November 1984; and in revised form 7 February 1985.

### Patients and methods

### *Patient 1*

A 54-year-old woman, gravida 7, para 4, was admitted to hospital in February 1983 with massive genital bleeding. Her last pregnancy occurred in 1977 and was a hydatidiform mole of the complete type. Hysterectomy revealed a uterine tumour diagnosed histologically as choriocarcinoma and in the absence of any teratomatous element. Urinary hCG at the time of operation was 102,400 IU $l^{-1}$.

### *Patient 2*

A 52-year-old woman, gravida 5, para 2, whose previous pregnancy was therapeutically aborted in 1980, presented in January 1984 when she had a urinary hCG of 128,000 iu $l^{-1}$. Operation and subsequent histopathology gave a diagnosis of choriocarcinoma.

Small blocks of tissue were taken at the time of operation and frozen either in a $-70°$ deep freeze (patient 1) or directly in liquid nitrogen. Frozen sections (5–8 $\mu$m) were cut, the sections air-dried, fixed in acetone for 10 min, air-dried further and then wrapped individually in aluminium foil so as to seal them.

Some of these sections were then sent at ambient temperature from Niigata, Japan, to Bristol, UK, where they were stored at $-20°$. Sections were stained with the monoclonal antibodies listed in Table I using the indirect immunoperoxidase technique according to previously published procedures (Sunderland *et al.*, 1981).

All antibodies were used at a predetermined, saturating dilution. The level of background staining on each cell type was established with the MA 2.1 antibody which did not react with uterus or

© The Macmillan Press Ltd., 1985

**Table I** Monoclonal antibodies used in the study

| *Monoclonal antibody* | *Specificity* | *Reference* | *Preparation* |
|---|---|---|---|
| W6/32 | Class I (HLA-A,B,C) heavy chain, monomorphic determinant[a] | Barnstaple *et al.* (1978) | Ascites |
| PA 2.6 | Class I (HLA-A,B,C) heavy chain, monomorphic determinant | Brodsky & Parham, (1982) | Immunoglobulin |
| BB 7.7 | Class I (HLA-A,B,C) intact molecule only, monomorphic determinant | Brodsky & Parham, (1982) | Immunoglobulin |
| BBM 1 | $\beta_2$ microglobulin, Class I antigen light chain, monomorphic determinant | Brodsky & Parham, (1982) | Immunoglobulin |
| MA 2.1 | Class I HLA A2 and B17 only, polymorphic determinant | McMichael *et al.*, (1980) | Ascites |
| ME 1 | Class I HLA B7, B22 and B27 only, polymorphic determinant | Eliss *et al.*, (1982) | Ascites |
| NFK 1 | Class II (HLA-DR) antigens, monomorphic determinant[b] | Fuggle *et al.* (1983) | Ascites |
| Anti Leu 10 | Class II (HLA-DC) antigens, monomorphic determinant | unpublished[c] | Immunoglobulin |
| F 10-89-4 | Leucocyte common antigen | Dalchau *et al.* (1980) | Immunoglobulin |
| Anti Leu M3 | Monocyte/macrophage antigen | Dimitriu-Bona *et al.* (1983) | Immunoglobulin |
| TROMA 1 | Specialised subgroup of intermediate filaments | Kemler *et al.* | Tissue cilture supernatant |
| NDOG1 | Villous syncytiotrophoblast plasma membrane, some non-villous trophoblast, unreactive with most adult tissues, including liver, kidney, heart, brain, colon, pancreas and pregnant uterus | Sunderland *et al.* (1981) | Tissue culture supernatant |
| NDOG2 | Placental alkaline phosphatase | Sunderland *et al.* (1984*b*) | Tissue culture supernatant |

[a]The term "monomorphic" means that the determinant recognised is common to all HLA antigens of this designation.
[b]The reactivity of this monoclonal antibody with the HLA DC and SB Class II locus products has not yet been determined.
[c]Unpublished data and antibody available from Becton–Dickinson.

tumour in either patient. Background staining was minimal in Patient 1 but a low level of diffuse activity from blood was present close to the site of tumour implantation in Patient 2.

To control for any selective loss of antigenicity during transport, sections from the same blocks of tissue were stained in Niigata using the W6/32 antibody to Class 1 (HLA A,B,C) antigens, a similar HLA-DR antibody (Cappel Co. USA), NDOG2 and TROMA 1 by the avidin-biotin technique. Identical results were obtained in Japan and in Bristol.

### Results

Frozen sections of choriocarcinoma tissue were stained with the panel of monoclonal antibodies listed in Table I using the indirect immunoperoxidase technique.

The antibody W6/32 detects a monomorphic determinant of Class I (HLA A,B,C) antigen molecules (i.e. it detects all molecules of this class). The antibody was found to stain a major population (40–70%) of choriocarcinoma cells in both tumours examined. The negative tumour cell population included some prominent groups of small cytotrophoblasts and some large syncytial giant cells. The staining pattern is illustrated in Figure 1a. Positively stained cells typically showed an outer ring of reactivity at the plasma membrane with a lesser reaction in the cytoplasm. Three other antibodies to monomorphic determinants of MHC Class I antigens gave identical staining patterns as judged by comparison of serial sections. These included antibodies recognising determinants on the heavy chain (W6/32, PA 2.6), the $\beta_2$-microglobulin light chain (BBM 1) and determinants generated by combination of the two polypeptide chains of the Class I molecule (BB 7.7) (Brodsky & Parham, 1982).

The antibody ME1 recognises a polymorphic determinant of the Class I molecule and specifically detects the HLA B7, B22 and B27 specificities. Conventional HLA typing of Patient 1 showed her to carry HLA A24/A31, B7/BW55. Figure 1b demonstrated a positive reaction of the ME1 antibody with the patient's uterine tissue as expected from her possession of HLA B7. In contrast the vast majority of tumour tissue did not react with ME1 (compare Figure 1*b* with 1*a*). Such unreactivity might be due to the fully allogenic status of this molar-derived tumour whereby it carries foreign, paternal HLA antigens. The patient's husband, however, typed as HLA A24/A26, B7/blank. The B locus blank was noted as possibly BW60 or BW48, but the probability remained that the husband was homozygous for HLA B7 and therefore that the tumour tissue was of the correct genotype for ME1 reactivity. Small foci of ME1 reactivity were, however, apparent in some areas of tumours. These cells occurred in groups and were of variable staining intensity (Figure 2), making up less than 10% of the total tumour cell population. They were of typical tumour cell morphology and were found amongst populations that were uniformly positive for the trophoblast marker, TROMA 1 on serial sections. The MA 2.1 antibody, specific for HLA A2 and B17, reacted with neither host nor tumour tissue in Patient 1. Patient 2 was HLA-typed as A10(A26)/AW33, B35/BW60 or BW61 and her husband as A2/A30 or A31, BW57/BW48. Neither ME1 nor MA2.1 were reactive with either host or tumour tissue in this patient.

MHC Class I antigens were absent from the vast majority of tumour cells (Figure 3). The small number of scattered DR positive cells seen within the tumour tissue may derive from the bank of such DR positive cells present in the uterine tissue immediately adjacent to the tumour implantation site (Figure 3). Very similar patterns of reactivity were found in and around such implantation sites with NFK-1, antiLeu 10, antiLeu M3 and FID-89-4 suggesting that the majority of these cells may be DR. DC positive macrophages.

Further heterogeneity of the tumour cells was demonstrated with monoclonal anti-trophoblast antibodies. Whilst TROMA 1 stained 90–100% of tumour cells in both patients, the antibodies NDOG1 and NDOG2 showed a considerable variability. In patient 1, NDOG1 reacted with ~50% of tumour cells and was conspicuous on groups of cells which did not express Class 1 antigen as assessed on serial sections. In patient 2 NDOG1 reacted with >80% of tumour cells and stained cells showed no obvious relation to Class 1 antigen expression. The antibody, NDOG2, which detects placental alkaline phosphatase, was entirely negative in patient 1, but detected a small group of large tumour cells in patient 2 which comprised <10% of the total.

### Discussion

Two major populations of choriocarcinoma cells have been distinguished on the basis of the expression of MHC Class I antigen monomorphic determinants (Figure 1a). Such antigens can also be detected on major subpopulations of proliferating extra-villous trophoblast in both the early human placenta and hydatidiform mole (Sunderland *et al.*, 1981; 1984). It is from such proliferating extra-villous trophoblast that choriocarcinoma is detected similarly by antibodies recognising heavy chain, light chain or only the intact molecule suggesting that a balanced, co-ordinated expression of Class I polypeptide chains predominates *in vivo*. In contrast, JaR choriocarcinoma cells in culture have been shown to express $\beta_2$-microglobulin in the absence of Class I heavy chain (Trowsdale *et al.*, 1980). In these respects the choriocarcinoma antigen is similar to that of the normal lymphocyte.

Recent studies of normal placental extra-villous trophoblast by Redman *et al.*, (1984) have demonstrated that MHC Class I antigens are expressed by trophoblast in a manner whereby only monomorphic but not polymorphic determinants are detectable and Redman has suggested that trophoblast MHC Class I antigen might be biochemically distinct from the lymphocyte antigen. Similar data have also been obtained for the extra-villous trophoblast of hydatidiform mole (Sunderland *et al.*, 1984). Although providing no definitive data, the present study on choriocarcinoma is also consistent with this idea. Thus the tumour carried by patient 1 derived from a complete hydatidiform mole and therefore carried only a paternal genotype (Jacobs *et al.*, 1980). HLA typing data showed the father to be probably homozygous B7 and thereby suggested the tumour carried the HLA B7 haplotype. This was further supported by the expression of an MEI-reactive antigen by a very small population of tumour cells (Figure 2). It is therefore possible that the lack of MEI reactivity by the vast majority of the cells which express MHC Class I monomorphic determinants (Figure 1) is indicative of a similar abnormality in choriocarcinoma trophoblast as has been found in normal and molar extra-villous trophoblast.

In addition to the two major choriocarcinoma cell populations distinguished by expression of MHC Class I monomorphic determinants (Figure 1*a*), we have also detected a very small population

**Figure 1** Gestational choriocarcinoma from Patient 1 was stained with monoclonal antibodies to Class I (HLA A,B,C) antigens using the indirect immunoperoxidase technique on frozen sections. (a) W6/32 monoclonal antibody stains maternal uterine tissue (U) and a major subpopulation of choriocarcinoma cells. A group of unstained small cytotrophoblast cells is arrowed. (×60). (b) ME1 monoclonal antibody specific for HLA B7, B22 and B27 stains the maternal uterine tissue (U) but not the tumour cells. (×60). Nuclei were counterstained with hematoxylin.

**Figure 2** An area of Patient 1 gestational choriocarcinoma tissue in which some cells appeared to express the polymorphic Class I antigen determinant detected by ME1 monoclonal antibody. A group of intensely reactive and a group of much more weakly reactive tumour cells are arrowed. ( × 190). Otherwise as **Figure 1.**

**Figure 3** Patient 1 gestational choriocarcinoma stained with NFK-1 monoclonal antibody directed to Class II (HLA DR antigens). Tumour tissue is predominantly unstained, but aggregates of reactive cells are apparent in the uterine tissue immediately adjacent to the site of tumour implantation. ( × 75). Otherwise as **Figure 1.**

(Figure 2) which appears to express polymorphic determinants. The conclusion, however, rests on the staining of a very few cells in only one case and must therefore be offered tentatively at this stage. Such cells have not been previously reported in normal or molar pregnancy although we have observed a few cells of this phenotype in anchoring villous cytotrophoblast present in a normal 12–16 week placenta obtained by pregnancy hysterectomy (Sunderland & Stirrat, unpublished data). It may be that it is this antigenically competent third population of cells which gives rise to the anti paternal antigen-specific antibodies characteristic of choriocarcinoma (Lawler *et al.*, 1976; Shaw *et al.*, 1979). Indeed since this placental tumour manifests no foetus, foetal circulation or placental villous stroma, it is difficult to conceive of any other immunogenic stimulus.

The heterogeneity of this tumour is well established by conventional histopathology. The patterns of staining obtained with the monoclonal antibodies employed here demonstrates a further dimension to this heterogeneity. This includes three populations defined by Class 1 antigen expression, another on the basis of NDOG1 antibody staining and a third minor population in terms of placental alkaline phosphatase expression. The clonal derivation of these cell populations is suggested by their tendency to occur in groups (eg Figure 1a).

We are grateful to Dr J.N. Bulmer for helpful discussion and to the following for the gift of monoclonal antibodies: Professor W. and Dr J. Bodmer, Dr. R. Kemler, Dr A.J. McMichael, Dr Sue Fuggle and Dr. A.F. Williams. This work was supported by MRC Grant No. 8120833 SB.

### References

BARNSTABLE, C.J., BODMER, W.F., BROWN, G. & 4 others. (1978). Production of monoclonal antibodies to group A erythrocytes, HLA and other human cell surface antigens. *Cell*, **14**, 9.

BRODSKY, F.M. & PARHAM, P. (1982). Monomorphic anti HLA A,B,C monoclonal antibodies detecting molecular subunits and combinatorial determinants. *J. Immunol.*, **128**, 129.

DALCHAU, R., KIRKLEY, J. & FABRE, J.W. (1980). Monoclonal antibody to a human leucocyte-specific membrane glycoprotein probably homologous to the leucocyte-common (L-C) antigen of the rat. *Eur. J. Immunol.*, **10**, 737

DIMITRIU-BONA, A., BURMESTER, G.R., WATERS, S.J, & WINCHESTER, J. (1983). Human mononuclear phagocyte differentiation antigens. I Patterns of antigenic 1. expression on the surface of human monocytes and macrophages defined by monoclonal antibodies. *J. Immunol.*, **130**, 145.

ELLIS, S.A., TAYLOR, C. & MCMICHAEL, A.J. (1982). Recognition of HLA B27 and related antigens by a monoclonal antibody. *Hum. Immunol.*, **5**, 49.

ELSTON, C.W. (1969). Cellular reaction to choriocarcinoma. *J. Pathol. Bacteriol.*, **97**, 261.

FUGGLE, S.V., ERRASTI, P., DAAR, A.S. FABRE, J.W., TING, A. & MORRIS, P.J. (1983). Localisation of major histocompatibility complex (HLA-ABC and DR) antigens in 46 kidneys. *Transplantation*, **35**, 385.

JONES, E.A. & BODMER, W.F. (1980). Lack of expression of HLA antigens on choriocarcinoma cell lines. *Tissue Antigens*, **16**, 195.

KEMLER, R., BRULET, P., SCHNEBELEN, M-T, & 2 others. (1981). Reactivity of monoclonal antibodies against intermediate filament proteins during embryonic development. *J. Embryol. Exp. Morphol.*, **64**, 45.

JACOBS, P.A., WILSON, C.M., SPRENKLE, J.A., ROSENSHEIN, N.B. & MIGEON, B.R. (1980). Mechanism of origin of complete hyadtidiform moles. *Nature*, **286**, 714.

LAWLER, S.D., KLOUDA, P.T. & BAGSHAWE, K.D. (1976). The relationship between HLA antibodies and the causal pregnancy in choriocarcinoma. *Br. J. Obstet. Gynecol.*, **83**, 651.

MCMICHAEL, A.J., PARHAM, P.R., RUST, N. & 1 other. (1980). A monoclonal antibody that recognises an antigenic determinant shared by HLA A2 and B17. *Hum. Immunol.*, **1**, 121.

REDMAN, C.W.G., MCMICHAEL, A.J., STIRRAT, G.M. & 2 others. (1984). Class I Major Histocompatibility Complex antigens on human extra-villous trophoblast. *Immunology*, **52**, 457.

SHAW, A.R.E., DASGUPTA, M.K., KOVITHAVONGS, T. & 4 others. (1979). Humoral and cellular immunity to paternal antigens in trophoblastic neoplasia. *Int. J. Cancer*, **24**, 586.

SUNDERLAND, C.A., DAVIES, J.O. & STIRRAT, G.M. (1985*a*). Immunohistology of normal and ovarian cancer tissue with a monoclonal antibody to placental alkaline phosphatase. *Cancer Res.* (in press).

SUNDERLAND, C.A., REDMAN, C.W.G. & STIRRAT, G.M. (1981). HLA A,B,C antigens are expressed on non-villous trophoblast of the early human placenta. *J. Immunol.*, **127**, 2614.

SUNDERLAND, C.A., REDMAN, C.W.G. & STIRRAT, G.M. (1985*b*). Characterisation and localisation of HLA antigens on hydatidiform mole. *Am. J. Obstet. Gynecol.* (in press).

TANAKA, K., NABESHIMA, Y., TAKAHASHI, TAKEUCHI, S., NABASHINA, Y. & OGATA, K. (1981). Lack of effective messenger RNA for beta$_2$-microglobulin in a gestational choriocarcinoma cell line (GCH-1). *Cancer Res.*, **41**, 3639.

TROWSDALE, J., TRAVERS, P., BODMER, W.F.& PATILLO, R.A. (1980). Expression of HLA A,B,C and B$_2$-microglobulin antigens in human choriocarcinoma cell lines. *J. Exp. Med.*, **152**, 11s.